# Warm weather increases emergency room visits

**DOI:** 10.1038/s43856-021-00064-6

**Published:** 2022-01-06

**Authors:** Katharine Barnes

**Affiliations:** Communications Medicine, https://www.nature.com/commsmed

## Abstract

High ambient temperatures are associated with adverse health effects and increased death. A recent article published in *The BMJ* found an association between extreme heat and an increase in the number of visits to the emergency department by adults in the USA.

**Figure:**
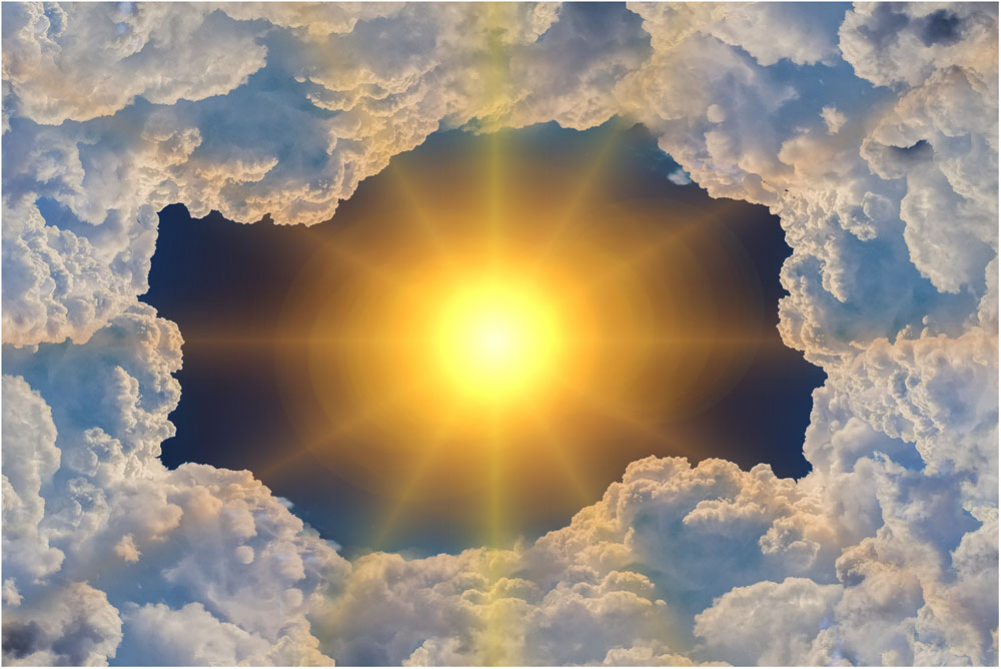
Pixabay

One projected impact of climate change is an increase in the number and intensity of days of extreme heat. Heat is known to increase renal, cardiorespiratory and mental illnesses in adults aged 65 and older, increasing both hospital admissions and death. Emergency department admissions might provide a more accurate measure of problematic symptoms than alternatives used previously. Also, few studies have investigated the impact of elevated ambient temperatures on rates of admissions to emergency departments or the impact on younger adults.

In this US-based study from Sun and colleagues, the risk of emergency department visit was estimated for more than 74 million adults who were commercial or Medicare Advantage beneficiaries^1^. The inclusion of people with health care funded by both commercial and Medicare systems enabled evaluation of the impact of heat on high and low income adults, as Medicare Advantage provides health care for patients unable to fund such care themselves. Over 21 million emergency department visits were recorded for these individuals between 2010 and 2019. Local maximum daily temperatures were used to define extreme heat for particular locations, which averaged 34.4 °C across the USA.

An association was observed between the daily temperature and the relative risk of emergency department visit. Separating the admissions by cause demonstrated that visits due to heat-related illness, renal disease and mental disorders increased. However, there was no association between heat and visits for cardiovascular or respiratory disease. The lack of association seen for cardiovascular admissions has been hypothesised to be due to people being more likely to die before being admitted to hospital in conditions of extreme heat.

The associations seen were strongest among young and middle-aged adults, and also more pronounced for men than women. A lower income was also shown to be associated with increased visits, regardless of ambient temperature. The authors hypothesise that occupational and recreational activities might be higher in those groups in which stronger associations were seen, although do not have any data on these characteristics.

The association between heat and emergency department visits was strongest in the north east, which has a cooler overall climate. In contrast, a weaker association was seen in the south east and areas with a tropical climate. This suggests either physiological or behavioural adaptations to heat might reduce risk of adverse effects to heat. This could potentially also explain why elderly people were less adversely impacted by excess heat, as there is greater public awareness of heat-related risks to the elderly. The information presented in this study should encourage there to be a greater awareness of the risks of excess heat on younger populations as well.
